# Current evidences on *XPC* polymorphisms and gastric cancer susceptibility: a meta-analysis

**DOI:** 10.1186/1746-1596-9-96

**Published:** 2014-05-23

**Authors:** Qiliu Peng, Zhiping Chen, Yu Lu, Xianjun Lao, Cuiju Mo, Ruolin Li, Xue Qin, Shan Li

**Affiliations:** 1 Department of Clinical Laboratory, First Affiliated Hospital of Guangxi Medical University, Nanning 530021, Guangxi, China; 2 Department of Occupational Health and Environmental Health, School of Public Health at Guangxi Medical University, Nanning, Guangxi, China; 3 Department of Medicine Research, First Affiliated Hospital of Guangxi Medical University, Nanning 530021, Guangxi, China

**Keywords:** XPC, Polymorphism, Gastric cancer, Meta-analysis

## Abstract

**Background:**

Reduced DNA repair capacities due to inherited polymorphisms may increase the susceptibility to cancers including gastric cancer. Previous studies investigating the association between *Xeroderma Pigmentosum group C* (*XPC*) gene polymorphisms and gastric cancer risk reported inconsistent results. We performed a meta-analysis to summarize the possible association.

**Methods:**

All studies published up to January 2014 on the association between *XPC* polymorphisms and gastric cancer risk were identified by searching electronic databases PubMed, EMBASE, Cochrane library, and Chinese Biomedical Literature database (CBM). The association between *XPC* polymorphisms and gastric cancer risk was assessed by odds ratios (ORs) together with their 95% confidence intervals (CIs).

**Results:**

Six studies with 1,355 gastric cancer cases and 2,573 controls were finally included in the meta-analysis. With respect to Lys939Gln polymorphism, we did not observe a significant association when all studies were pooled into the meta-analysis. When stratified by ethnicity, source of control, and study quality, statistical significant association was not detected in all subgroups. With respect to Ala499Val and PAT−/+polymorphisms, we also did not observe any significant association with gastric cancer risk in the pooled analysis.

**Conclusions:**

This meta-analysis based on current evidences suggested that the *XPC* polymorphisms (Lys939Gln, Val499Arg, and PAT−/+) did not contribute to gastric cancer risk. Considering the limited sample size and ethnicity included in the meta-analysis, further larger scaled and well-designed studies are needed to confirm our results.

**Virtual Slides:**

The virtual slide(s) for this article can be found here: http://www.diagnosticpathology.diagnomx.eu/vs/1485880312555069

## Background

Gastric cancer is one of the most common cancers and cancer related deaths are highly prevalent worldwide [1]. The development of gastric cancer is a multifactorial and multistep process. Previous epidemiological investigations have identified that high consumption of salty food, low consumption of fresh fruits and vegetables, smoking, drinking, and Helicobacter pylori infection were the major contributors to the development and progression of gastric cancer [2-4]. However, most individuals exposed to these environmental risk factors never develop gastric cancer while many gastric cancer cases develop among individuals without those environmental factors, suggesting that other factors such as genetic factors also play important roles in gastric carcinogenesis.

The DNA repairing system, which was composed of many DNA repair genes, plays an important role in removing damaged genes, maintaining the genomic integrity and preventing carcinogenesis. The *xeroderma pigmentosum complementation group C* (*XPC*) is one of the eight core genes (i.e., ERCC1, XPA, XPB, XPC, XPD, XPE, XPF, and XPG) in the nuclear excision repair (NER) pathway of the DNA repairing system. XPC binds to HR23B and forms the XPC-HR23B complex, which is involved in the DNA damage recognition and DNA repair initiation in the NER pathway [5-7], and the binding of XPC to damaged DNA is the rate-limiting step for NER [8]. The *XPC* gene, which encodes a 940-amino acid protein, spans 33 kb on chromosome 3p25 and contains 16 exons and 15 introns [9]. There are at least 687 reported single nucleotide polymorphisms (SNPs) in the *XPC* gene region (http://www.ncbi.nlm.nih.gov/projects/SNP). Among all the identified SNPs, three common polymorphisms have been extensively studied: (a) a substitution of alanine for valine in codon 499 (Ala499Val, rs2228000), in the interaction domain of XPC with hHRAD23; (b) an A to C transversion in exon 15 resulting in a lysine-to-glutamine transition at position 939 (Lys939Gln, rs2228001), located in the interaction domain with TFIIH; and (c) a poly AT region on intron 9 (PAT−/+). It was reported that the variant alleles of the three polymorphisms in the *XPC* genes correlated with relatively high DNA adduct levels in lymphocyte DNA, indicating that these polymorphisms were associated with decreased DNA repair capacity [10,11]. Therefore, it was biologically reasonable to hypothesize a potential relationship between the *XPC* gene polymorphisms and cancer susceptibility.

To date, the *XPC* gene polymorphisms (Lys939Gln, Val499Arg, and PAT−/+) to gastric cancer risk have been a research focus in scientific community and have drawn increasing attention. Several original studies have reported the role of *XPC* polymorphisms in gastric cancer risk [12-17], but the results are inconclusive. For genetic association studies that investigate candidate polymorphisms, sample size is an important influencing factor for study accuracy. Small sample size might have inadequate power to explore a true association of modest effect [18], especially for complex multifactorial disease such as gastric cancer. While combining data from all eligible studies by meta-analysis has the advantage of increasing statistical power and reducing random error and obtaining precise estimates for some potential genetic associations. Therefore, in this study, we conducted a quantitative meta-analysis including all eligible studies.

## Methods

### Search strategy

We conducted a comprehensive literature search in PubMed, EMBASE, Cochrane library, and Chinese Biomedical Literature (CBM) databases up to January 01, 2014 using the following search strategy: (“gastric cancer”) and (“xeroderma pigmentosum complementation group C”, or “XPC”). There was no restriction on time period, sample size, population, language, or type of report. All eligible studies were retrieved and their references were checked for other relevant studies. The literature retrieval was performed in duplication by two authors independently (Qiliu Peng and Yu Lu). When multiple publications reported on the same or overlapping data, we chose the most recent or largest population.

### Inclusion and exclusion criteria

Studies were considered if they met the following inclusion criteria: (1) case–control or cohort studies which evaluated the association between *XPC* polymorphisms and gastric cancer; (2) had an odds ratio (OR) with 95% confidence interval (CI) or other available data for estimating OR (95% CI); and (3) the control population did not contain malignant tumor patients. Studies were excluded if one of the following existed: (1) no control population; (2) duplicate of previous publication; and (3) insufficient information for data extraction.

### Data extraction

Information was extracted by two authors independently according to the inclusion criteria listed above. Data extracted from eligible studies included the first author, publication year, ethnicity, country, genotyping methods, matching criteria, source of control, gastric cancer ascertainment, total numbers of cases and controls and genotype frequencies of cases and controls. Ethnic backgrounds were categorized as Caucasian, Asian. To ensure the accuracy of the information extracted, the two authors checked the data extraction results and reached consensus on all of the items. If different results generated, they would check the data again and have a discussion to come to an agreement. If these two authors could not reach a consensus, another author (Xue Qin) was consulted to resolve the dispute.

### Quality score assessment

The quality of eligible studies was assessed independently by two authors (Qiliu Peng and Xianjun Lao) according to a set of predefined criteria (Table 1) modified from our previous meta-analysis of molecular association study [19]. The revised criteria cover the representativeness of cases, source of controls, ascertainment of gastric cancer, total sample size, quality control of genotyping methods, and Hardy-Weinberg equilibrium (HWE) in the control population. Disagreements were resolved by consensus. Total scores ranged from 0 (lowest) to 10 (highest). Articles with scores equal to or higher than 7 were considered “high-quality” studies, whereas those with scores less than 7 were considered “low-quality” studies.

**Table 1:** Scale for quality assessment

**Criteria**	**Score**
Representativeness of cases	
Selected from cancer registry or multiple cancer center sites	2
Selected from oncology department or cancer institute	1
Selected without clearly defined sampling frame or with extensive inclusion/exclusion criteria	0
Source of controls	
Population or community based	2
Both population-based and hospital-based/healthy volunteers/blood donors	1.5
Hospital-based controls without gastric cancer	1
Cancer-free controls without total description	0.5
Not described	0
Ascertainment of gastric cancer	
Histologically or pathologically confirmed	2
Diagnosis of gastric cancer by patient medical record	1
Not described	0
Sample size	
>1000	2
200-1000	1
<200	0
Quality control of genotyping methods	
Clearly described a different genotyping assay to confirm the data	1
Not described	0
Hardy-Weinberg equilibrium	
Hardy-Weinberg equilibrium in controls	1
Hardy-Weinberg disequilibrium in controls	0.5
No checking for Hardy-Weinberg disequilibrium	0

### Statistical analysis

Crude odds ratios (ORs) with their 95% confidence intervals (CIs) were used to assess the strength of association between the *XPC* polymorphisms and gastric cancer risk. The pooled ORs were performed for co-dominant genetic models, dominant genetic model, and recessive genetic model, respectively. Heterogeneity among studies was checked by the chi-square-based Q-test and I^2^ statistics [20,21]. If the result of the heterogeneity test was P_Q_ < 0.1 or I^2^ ≥ 50%, indicating the presence of heterogeneity, a random-effects model (the DerSimonian and Laird method) was used to estimate the summary ORs [22]; otherwise, when the result of the heterogeneity test was P_Q_ ≥ 0.1 and I^2^ < 50%, indicating the absence of heterogeneity, the fixed-effects model (the Mantel–Haenszel method) was used [23]. Subgroup analyses were performed according to ethnicity, source of control, and study quality. Sensitivity analysis was conducted by sequential omission of individual study to assess the robustness of the results. Publication bias was assessed using a Begg’s funnel plot and Egger’s regression asymmetry test [24]. If publication bias existed, the Duval and Tweedie non-parametric “trim and fill” method was used to adjust for it [25]. The distribution of the genotypes in the control population was tested for HWE using a goodness-of-fit Chi-square test. All the statistical tests were performed using Stata software, version 12.0 (Stata Corp., College Station, TX).

## Results

### Eligible studies

Based on the search criteria, seven potential relevant studies were identified. One of these articles was excluded because it contained overlapping data [26]. Manual search of references cited in the published studies did not reveal any additional articles. As a result, a total of six studies met the inclusion criteria were included in the meta-analysis [12-17]. The main characteristics of the eligible studies were presented in Table 2. Among them, five studies [12,14-17] including 1,049 cases and 2,026 controls were available for Lys939Gln polymorphism, three studies [14,15,17] with 817 cases and 1438 controls for Val499Arg polymorphism, and two studies [13,15] containing 559 cases and 1159 controls for PAT−/+polymorphism. The sample size of these studies varied considerably, ranging from 222 to 977 individuals. Of all the eligible studies, two were conducted in Caucasians [12,16] and three were in Asians [14,15,17] for Lys939Gln polymorphism; all the three eligible studies for Val499Arg polymorphism were conducted in Asians [14,15,17]; one was conducted in Caucasians [13] and one [15] was in Asians for PAT−/+polymorphism. Three studies were population–based and three were hospital–based studies. One study in the present meta-analysis did not provide definite criteria for the CRC ascertainment [12]. Two genotyping methods were used, including PCR-RFLP and TaqMan assay. The genotype distributions of the controls were consistent with HWE in all of the included studies.

**Table 2:** Characteristics of studies included in the meta-analysis

**First author (Year)**	**Country**	**Ethnicity**	**Sample size (case/control)**	**Genotyping methods**	**Matching criteria**	**Source of control**	**GC ascertainment**	**SNPs**	**HWE(*P *** **value)**			**Quality scores**
									**Lys939Gln**	**Ala499Val**	**PAT+/−**	
Palli 2010	Italy	Caucasian	306/547	TaqMan	Gender	PB	Histo-	PAT+/−	—	—	0.085	7
Dong 2008	China	Asian	253/612	PCR-RFLP	Age and gender	PB	Histopatho-	Lys939Gln, Ala499Val, PAT+/−	0.699	0.217	0.786	8.5
Engin 2011	Turkey	Caucasian	106/116	PCR-RFLP	Age and BMI	HB	NR	Lys939Gln	0.642	—	—	4.5
Long 2010	China	Asian	361/616	TaqMan	Age, gender, ethnicity, smoking, and drinking	HB	Histopatho-	Lys939Gln, Ala499Val	0.446	0.673	—	6
Ye 2006	Sweden	Caucasian	126/472	PCR-RFLP	NR	PB	Histo-	Lys939Gln	0.540	—	—	7
Li 2010	China	Asian	203/210	PCR-RFLP	Age, gender, smoking, and drinking	HB	Patho-	Lys939Gln, Ala499Val	0.173	0.462	—	7.5

GC, Gastric cancer; Histopatho-, Histopathologically confirmed; Histo-, Histologically confirmed; Patho-, Pathologically confirmed; NR, Not reported; PB, Population–based; HB, Hospital–based; HWE, Hardy–Weinberg equilibrium in control population; PCR–RFLP, Polymerase chain reaction-restriction fragment length polymorphism; BMI, Body mass index.

### Quantitative analysis

#### *XPC* Lys939Gln

**Figure 1:**
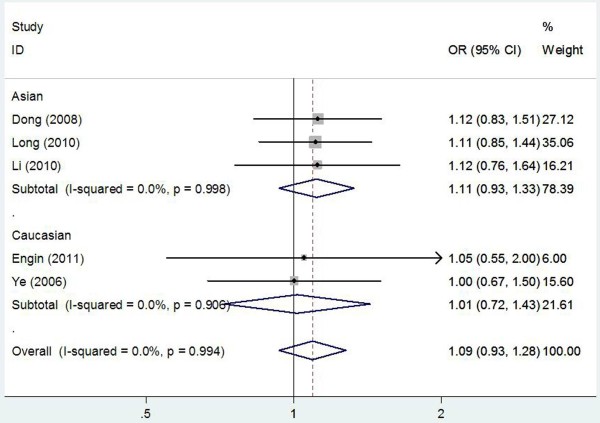
**Forest plot of subgroup analysis by ethnicity on the association between *XPC *** **Lys939Gln polymorphism and gastric cancer risk using a fixed-effect model (dominant model Gln/Gln + Gln/Lys vs. Lys/lys).**

The main results of meta-analysis of *XPC* Lys939Gln polymorphism and gastric cancer risk were present in Table 3. There was no evidence of significant association between *XPC* Lys939Gln polymorphism and gastric cancer risk when all the eligible studies were pooled into the meta-analysis (Gln/Gln vs. Lys/lys: OR = 1.123, 95% CI = 0.881–1.431, P = 0.349; Gln/Lys vs. Lys/lys: OR = 1.083, 95% CI = 0.917–1.277, P = 0.347; Gln/Gln + Gln/Lys vs. Lys/lys: OR = 1.092, 95% CI = 0.933–1.277, P = 0.273, Figure 1; Gln/Gln vs. Gln/Lys + Lys/lys: OR = 1.046, 95% CI = 0.838-1.307, P = 0.691). In subgroup analyses stratified by ethnicity, source of control, and study quality, statistically significant association was also not observed in all subgroups. Meanwhile, no significant heterogeneity was found in the pooled analysis and subgroup analyses (P_Q_ Values <0.1 and I^2^ > 50%).

**Table 3:** Meta-analysis of the XPC polymorphisms and gastric cancer risk

**Comparison**	**Population**	**No. of studies**	**Test of association**			**Mode**	**Test of heterogeneity**		
			**OR**	**95% CI**	***P *** **Value**		***χ***^***2***^	***P***_***Q ***_**Value**	***I***^***2***^
Lys939Gln									
Gln/Gln vs. Lys/lys	Overall	5	1.123	0.881-1.431	0.349	F	2.69	0.612	0.0
	Caucasian	2	1.016	0.490-2.109	0.965	F	2.31	0.128	46.8
	Asian	3	1.140	0.857-1.516	0.368	F	0.34	0.843	0.0
	PB	2	1.260	0.877-1.810	0.212	F	0.24	0.623	0.0
	HB	3	1.026	0.741-1.420	0.879	F	1.76	0.414	0.0
	High quality	3	1.179	0.860-1.616	0.308	F	0.75	0.686	0.0
	Low quality	2	1.048	0.718-1.530	0.807	F	1.71	0.191	41.6
Gln/Lys vs. Lys/lys	Overall	5	1.083	0.917-1.277	0.347	F	1.21	0.876	0.0
	Caucasian	2	0.993	0.689-1.432	0.972	F	0.87	0.352	0.0
	Asian	3	1.107	0.919-1.332	0.284	F	0.08	0.962	0.0
	PB	2	1.026	0.795-1.324	0.846	F	0.64	0.424	0.0
	HB	3	1.126	0.906-1.400	0.285	F	0.27	0.872	0.0
	High quality	3	1.062	0.855-1.319	0.587	F	0.90	0.639	0.0
	Low quality	2	1.112	0.861-1.437	0.417	F	0.24	0.628	0.0
Gln/Gln + Gln/Lys vs. Lys/lys	Overall	5	1.092	0.933-1.277	0.273	F	0.24	0.994	0.0
	Caucasian	2	1.015	0.720-1.430	0.933	F	0.01	0.906	0.0
	Asian	3	1.113	0.933-1.327	0.235	F	0.00	0.998	0.0
	PB	2	1.076	0.846-1.369	0.551	F	0.18	0.667	0.0
	HB	3	1.103	0.897-1.356	0.351	F	0.03	0.986	0.0
	High quality	3	1.087	0.886-1.333	0.424	F	0.21	0.900	0.0
	Low quality	2	1.098	0.860-1.401	0.453	F	0.02	0.881	0.0
Gln/Gln vs. Gln/Lys + Lys/lys	Overall	5	1.046	0.838-1.307	0.691	F	6.57	0.160	39.2
	Caucasian	2	0.921	0.344-2.468	0.870	F	5.98	0.114	43.3
	Asian	3	1.083	0.829-1.417	0.558	F	0.47	0.791	0.0
	PB	2	1.253	0.896-1.751	0.187	F	0.77	0.381	0.0
	HB	3	0.914	0.680-1.229	0.552	F	3.90	0.142	48.8
	High quality	3	1.157	0.862-1.553	0.331	F	1.66	0.435	0.0
	Low quality	2	0.828	0.400-1.712	0.610	F	3.90	0.148	44.3
Ala499Val									
Val/Val vs. Ala/Ala	Asian	3	0.861	0.632-1.172	0.341	F	0.57	0.754	0.0
Val/Ala vs. Ala/Ala	Asian	3	0.815	0.611-1.089	0.167	F	4.72	0.194	37.6
Val/Val + Val/Ala vs. Ala/Ala	Asian	3	0.823	0.630-1.076	0.155	F	4.49	0.106	35.5
Val/Val vs. Val/Ala + Ala/Ala	Asian	3	0.947	0.703-1.274	0.717	F	0.04	0.981	0.0
PAT−/+									
+/+vs. −/−	Overall	2	1.035	0.763-1.404	0.825	F	0.12	0.726	0.0
+/− vs. −/−	Overall	2	0.855	0.681-1.073	0.176	F	0.00	0.975	0.0
(+/+) + (+/−) vs. (−/−)	Overall	2	0.896	0.724-1.111	0.317	F	0.05	0.830	0.0
(+/+) vs. (+/−) + (−/−)	Overall	2	1.141	0.871-1.495	0.338	F	0.20	0.652	0.0

OR, odds ratio; CI, confidence intervals; R, random effects model; F, fixed effects model; PB, Population–based; HB, Hospital–based.

#### *XPC* Val499Arg

The main results of meta-analysis of *XPC* Val499Arg polymorphism and gastric cancer risk were summarized in Table 3. We did not found any significant association between *XPC* Val499Arg polymorphism and gastric cancer risk when all the eligible studies were pooled into the meta-analysis (Val/Val vs. Ala/Ala: OR = 0.861, 95% CI = 0.632–1.172, P = 0.341; Val/Ala vs. Ala/Ala: OR = 0.815, 95% CI = 0.611–1.089, P = 0.167; Val/Val + Val/Ala vs. Ala/Ala: OR = 0.823, 95% CI = 0.630–1.076, P = 0.155; Val/Val vs. Val/Ala + Ala/Ala: OR = 0.947, 95% CI = 0.703-1.274, P = 0.717). In addition, no significant heterogeneity was found in the pooled analysis (P_Q_ Values <0.1 and I^2^ > 50%). We did not perform subgroup analysis because of the limited number of studies available for *XPC* Val499Arg polymorphism.

#### *XPC* PAT−/+

The results of meta-analysis of *XPC* PAT−/+polymorphism and gastric cancer risk were summarized in Table 3. Overall, there were no significant associations of *XPC* PAT−/+polymorphism and gastric cancer risk when all the eligible studies were pooled into the meta-analysis (+/+vs. −/−: OR = 1.035, 95% CI = 0.763–1.404, P = 0.825; +/− vs. −/−: OR = 0.855, 95% CI = 0.681–1.073, P = 0.176; (+/+) + (+/−) vs. (−/−): OR = 0.896, 95% CI = 0.724–1.111, P = 0.317; (+/+) vs. (+/−) + (−/−): OR = 1.141, 95% CI = 0.871-1.495, P = 0.338). Moreover, statistical significant heterogeneity was also not found in the pooled analysis. We also did not perform subgroup analysis because only two studies were available for *XPC* PAT−/+polymorphism.

### Sensitivity analysis

**Figure 2:**
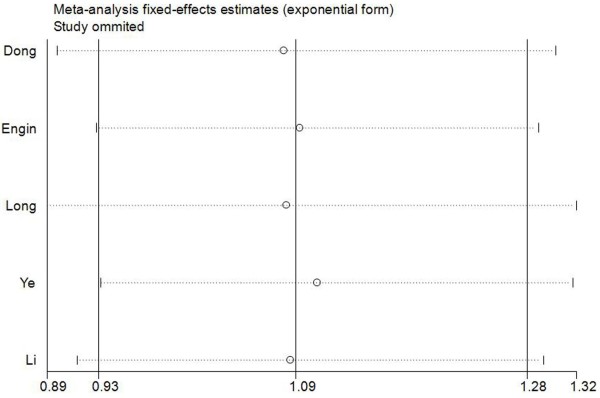
**Sensitivity analysis for XPC Lys939Gln polymorphism and gastric cancer risk (dominant model Gln/Gln + Gln/Lys vs. Lys/lys).** This figure shows the influence of individual studies on the summary OR. The middle vertical axis indicates the overall OR and the two vertical axes indicate its 95% CI. Every hollow round indicates the pooled OR when the left study is omitted in this meta-analysis. The two ends of every broken line represent the 95% CI.

A single study involved in the meta-analysis was deleted each time to reflect the influence of the individual data-set to the pooled OR, and the corresponding pooled OR was not materially altered (Figure 2), indicating that our results were statistically robust.

### Publication bias

Begger’s funnel plot and Egger’s test were performed to assess the publication bias of the included studies for *XPC* Lys939Gln and *XPC* Val499Arg polymorphisms. The shape of the funnel plot did not reveal any evidence of obvious asymmetry. Then, the Egger’s test was used to provide statistical evidence of funnel plot symmetry. The results still did not suggest any evidence of publication bias for *XPC* Lys939Gln (P = 0.152 for Gln/Gln vs. Lys/lys; P = 0.778 for Gln/Lys vs. Lys/lys; P = 0.339 for Gln/Gln + Gln/Lys vs. Lys/lys; P = 0.282 for Gln/Gln vs. Gln/Lys + Lys/lys) and *XPC* Val499Arg (P = 0.948 for Val/Val vs. Ala/Ala; P = 0.959 for Val/Ala vs. Ala/Ala; P = 0.937 for Val/Val + Val/Ala vs. Ala/Ala; P = 0.852 for Val/Val vs. Val/Ala + Ala/Ala). We did not carry out Begger’s funnel plot and Egger’s test for *XPC* PAT−/+polymorphism because only two studies were available for this polymorphism.

## Discussion

Previous studies evaluating the association between *XPC* polymorphisms (Lys939Gln, Val499Arg, and PAT−/+) and gastric cancer risk have provided inconsistent results, and most of these studies involved no more than a few hundred gastric cancer cases, which is too few to assess any genetic effects reliably. Meta-analysis has been recognized as an important way to detect the effect of selected genetic polymorphisms on disease risk precisely and to identify potential important sources of between-study heterogeneity. Hence, we performed this meta-analysis including all published studies to investigate the association between the *XPC* polymorphisms and gastric cancer risk. To the best of our knowledge, this is the first comprehensive meta-analysis of genetics studies on the association between *XPC* polymorphisms and gastric cancer risk. Our results suggested that the *XPC* polymorphisms (Lys939Gln, Val499Arg, and PAT−/+) were not associated with gastric cancer risk when all studies were pooled together. In subgroup analyses stratified by ethnicity, source of control, and study quality, statistical significant association was also not observed in all subgroups.

DNA repair mechanisms are important pathways in the removal of DNA adducts from damaged genomic sites and play crucial roles in the carcinogenesis of cancers [27]. XPC is a key member of the NER pathway. It binds to HR23B and forms the stable XPC-HR23B complex and is involved in the recognition and initiation of the genome repair of the NER pathway [5-7]. Mutations of the *XPC* genes may increase gastric cancer susceptibility by causing a severe depression of NER and consequently altering DNA repair activity [28]. However, in our study, no significant association between variant genotypes of *XPC* polymorphisms and gastric cancer risk was observed in the pooled analysis and subgroup analyses, which was inconsistent with the hypothesis above. Several potential concerns should be discussed for the non-significant associations between *XPC* polymorphisms and gastric cancer susceptibility. First, gastric cancer is a multi-factorial disease resulting from complex interactions between environmental and genetic factors [29,30]. It is possible that the variants at this locus have some modest effects on gastric cancer. Environmental factors, such as living habits and exposure to carcinogens, however, may also play a role in gastric cancer development. Thus, no regard of these factors may confer the non-significance for the independent role of *XPC* polymorphisms in gastric cancer development. Second, there are eight core genes (i.e., ERCC1, XPA, XPB, XPC, XPD, XPE, XPF, and XPG) in the NER pathway of the DNA repairing system, variants in these genes may interfere with each other in associated functions. This could cover the true associations of *XPC* gene polymorphisms with gastric cancer. Therefore, other variants as gastric cancer risk factors should be induced as co-variants to determine their true effects. The lack of considering above confounding factors might affect the significance of their results. Moreover, the null result may be due to the limited number of studies included in the meta-analysis, which had insufficient statistical power to detect a slight effect or may have generated a fluctuated risk estimate. Therefore, the negative results of the association between *XPC* polymorphisms and gastric cancer risk should be interpreted with caution.

Some limitations of this meta-analysis should be acknowledged. First, only published studies were included in the meta-analysis. It is possible that some related unpublished studies that might meet the inclusion criteria were missed; therefore, publication bias may have been present, even though statistical analysis indicated this not to be the case. Second, our results were based on unadjusted estimates and a more precise analysis could be conducted if more individual data were available; this would allow for adjustment by other covariates such as the quantity of salty food consumption, drinking, smoking and Helicobacter pylori infection; Third, in the pooled analyses for *XPC* Val499Arg and *XPC* PAT−/+polymorphisms, the number of studies included was relatively small, not having enough statistical power to investigate a real association of the polymorphisms with gastric cancer susceptibility. However, our meta-analysis also had some advantages. First, a substantial number of cases and controls were pooled from different studies, which significantly increased the statistical power compared with the individual studies. Second, no heterogeneity and publication bias was detected in our meta-analysis, indicating that the pooled results were precise and reliable.

## Conclusions

In conclusion, the result of this meta-analysis based on current evidences suggests that the *XPC* polymorphisms (Lys939Gln, Val499Arg, and PAT−/+) may not contribute to gastric cancer risk. However, it is necessary to conduct large sample studies using standardized unbiased genotyping methods, homogeneous gastric cancer patients, and well-matched controls. Moreover, gene–gene and gene–environment interactions should also be considered in the analysis. Such studies taking these factors into account may eventually lead to our better, comprehensive understanding of the association between the *XPC* polymorphisms and gastric cancer risk.

## Abbreviations

HWE: Hardy–Weinberg equilibrium; XPC: Xeroderma Pigmentosum group C; NER: Nuclear excision repair; SNP: Single nucleotide polymorphism; OR: Odds ratio; CI: Confidence interval.

## Competing interest

The authors declare that they have no competing interest.

## Authors’ contributions

QP, XQ performed the literature search, data extraction, statistical analysis and drafted the manuscript. QP, YL, XL, CM, and RL participated in data extraction. SL, XQ, ZC supervised the literature search, data extraction, statistical analysis and drafted the manuscript. All authors read and approved the final manuscript.
